# Potential Screening at Electrode/Ionic Liquid Interfaces from In Situ X‐ray Photoelectron Spectroscopy

**DOI:** 10.1002/open.201900211

**Published:** 2019-09-19

**Authors:** Francesco Greco, Sunghwan Shin, Federico J. Williams, Bettina S. J. Heller, Florian Maier, Hans‐Peter Steinrück

**Affiliations:** ^1^ Lehrstuhl für Physikalische Chemie 2 Friedrich-Alexander-Universität Erlangen-Nürnberg Egerlandstr. 3 91058 Erlangen Germany; ^2^ Departamento de Química Inorgánica, Analítica y Química Física, Facultad de Ciencias Exactas y Naturales, INQUIMAE-CONICET Universidad de Buenos Aires, Ciudad Universitaria Pabellón 2 Buenos Aires C1428EHA Argentina

**Keywords:** ionic liquids, photoelectron spectroscopy, potential screening, binding energies, electrodes

## Abstract

A new approach to investigate potential screening at the interface of ionic liquids (ILs) and charged electrodes in a two‐electrode electrochemical cell by in situ X‐ray photoelectron spectroscopy has been introduced. Using identical electrodes, we deduce the potential screening at the working and the counter electrodes as a function of applied voltage from the potential change of the bulk IL, as derived from corresponding core level binding energy shifts for different IL/electrode combinations. For imidazolium‐based ILs and Pt electrodes, we find a significantly larger potential screening at the anode than at the cathode, which we attribute to strong attractive interactions between the imidazolium cation and Pt. In the absence of specific ion/electrode interactions, asymmetric potential screening only occurs for ILs with different cation and anion sizes as demonstrated for an imidazolium chloride IL and Au electrodes, which we assign to the different thicknesses of the electrical double layers. Our results imply that potential screening in ILs is mainly established by a single layer of counterions at the electrode.

## Graphical abstract

**Potential screening** at the interface of ionic liquids and charged electrodes has been studied using a two‐electrode electrochemical cell by in situ X‐ray photoelectron spectroscopy. We found that the magnitude of potential screening strongly depends on the material of the electrode and the properties of the counterion.

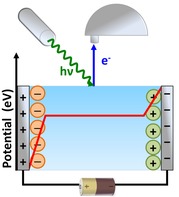

Ionic liquids (ILs) have drawn significant interest in electrochemistry due to their enormous potential as solvent‐free electrolytes for applications in batteries, supercapacitors, and electrodeposition.^1^ Understanding their properties at charged interfaces is crucial for applications because many physicochemical phenomena occur at the interface like capacitance charging and redox reactions. The interfacial properties have been addressed by electrochemical impedance spectrometry (EIS),^2^ cyclic voltammetry (CV),^3^ sum‐frequency generation (SFG),^4^ X‐ray reflectivity,^5^ scanning tunneling microscopy,^6^ atomic force microscopy (AFM),^7^ infrared,^8^ Raman,^9^ and nuclear magnetic resonance (NMR) spectroscopy.^10^ Also, new theoretical approaches, such as the Kornyshev model and computational simulations have been used to study the electrical double layer (EDL).^11^ These studies reveal various unique properties of the EDL at the IL/electrode interface, e. g., the slow response of ions,^12^ hysteresis behavior,^13^ layering,^14^ and bell and camel shape of differential capacitance curves.^15^

Within the last decade, X‐ray photoelectron spectroscopy (XPS) has been increasingly used to study surface/interface‐related phenomena in ILs, which is possible due to their negligible vapor pressure.^16^ With variable depth information in the nm range, angle‐resolved XPS has been successfully utilized to characterize liquid/solid, liquid/vacuum interfaces, and surface reactions.^17^ Recently, charged IL interfaces have been studied by monitoring charging shifts at the IL/vacuum interface by XPS. This approach can visualize the potential screening across the electrode/IL interface with the slow dynamic response of ILs to an applied voltage.^18^

The EDL structure and properties at charged IL/electrode interfaces are influenced by asymmetric properties of cations and anions, such as size, shape, dielectric constants, and specific interactions with the electrode. In addition, the polarity of the electrode can affect the EDL, due to different counterions at anode and cathode. Recently, the reliability of experimental observations of charged IL interfaces by EIS and CV has been discussed. These techniques can yield inconsistent/irreproducible results, due to impurities, neglect of the slow kinetic response, and questionable data analysis in EIS.3a, 11b, 15a, 19

Herein, we systematically study the properties of EDLs using XPS, which provides complementary and reliable information on the asymmetric potential screening at various IL/electrode interfaces (ILs studied, see Table S1 in the Supporting Information). Our approach has various advantages: (1) we measure potential screening in an equilibrium state after the formation of the EDL, thus slow kinetics of the ions is not affecting our results. (2) we measure under ultra‐high vacuum conditions with very clean ILs, as was carefully checked by XPS.^20^ (3) XPS gives direct access to the potential screening, without having to assume an equivalent circuit in EIS, which has been questioned in literature.^19^ The properties of the EDLs can be directly studied by comparing the potential screening on anode and cathode via binding energy shifts of IL signals in XPS. We believe that these advantages open a new route to address IL/electrode interfaces by systematically studying various ILs and electrodes.

Figure 1a shows our electrochemical setup for XPS at the IL/vacuum interface. The external voltages are applied to the ILs through identical (same material and contact area) working and counter electrodes (WE and CE), of which one was connected to ground together with the electron analyzer. The applied voltage is screened by the counterions of the IL through the formation of EDLs at both IL/electrode interfaces. Hence, the potential of the IL, that is, all core levels and the vacuum level, shifts by the amount of screening at the grounded electrode; see Figure 1b. The XP spectra were measured once the potential screening reached a steady‐state, as verified by chronoamperometry (Figure S2).

**Figure 1:**
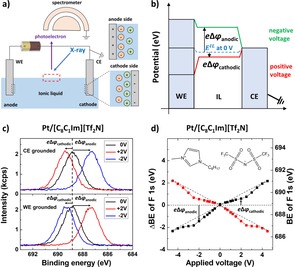
a) Schematic figure of our experimental setup and the charged interfaces with the CE grounded. b) Potential diagram of IL and electrodes with an applied positive (red) or negative (green) potential. The potential drops at the CE/IL interface are indicated as Δφ_anodic_ and Δφ_cathodic_ according to the polarity of the electrodes. c) F 1s spectra of [C_8_C_1_Im][Tf_2_N] with two identical Pt electrodes. d) ΔBE of the F 1s peak of [C_8_C_1_Im][Tf_2_N] vs. applied potential with CE grounded (black) and WE grounded (red). The ideal lines for equal potential drops at the anode/cathode interfaces are indicated as dashed straight lines (±0.5 eV/V), inset: molecular structure of [C_8_C_1_Im][Tf_2_N].

The measured binding energy (*BE*) of the IL XPS peaks is defined based on the usual convention as:

$$BE=h\nu -{KE}^{SP}-\phi^{SP}$$

where hν is the photon energy and *KE* ^*SP*^ is the electron kinetic energy at the spectrometer with the work function *φ* ^*SP*^. With the CE grounded, BE is affected by the vacuum level of the IL and thus the amount of potential screening Δ*ϕ* ^*CE/IL*^ between CE and IL can be determined by the BE shift (ΔBE ):

$$\Delta BE={e\Delta \varphi }^{CE/IL}$$

Detailed relations are described in the Supporting Information.

Figure 1c (top) shows the F 1s spectra of [C_8_C_1_Im][Tf_2_N] for different voltages applied between two Pt electrodes with the CE grounded. For 0 V, the F 1s peak is observed at 688.85 (±0.05) eV. When +2 V is applied to the WE, the F 1s signal shifts to larger *BE* by Δ*BE*=0.55 eV. This shift is due to the fact that for a positive applied voltage at the WE, the CE is charged negatively, and the potential of the IL decreases by the amount of screening, which is induced at the CE/IL (cathode) interface; consequently, Δ*BE* indicates the amount of potential screening at the cathode/IL interface. At −2 V the F 1s peak is shifted to lower *BE* by Δ*BE*=−1.45 eV. With a negative applied voltage, the CE is charged positively and the *BE* shifts by the amount of potential screening at the CE/IL (anode) interface, defined as “anodic voltage” (Δ*ϕ*_anodic_). Notably, charging effects due to X‐ray irradiation were negligible (<0.05 eV for 3000 s), as expected from a previous study.^21^

Within the electrochemical window of the IL, the applied voltage is completely screened at the two IL/electrode interfaces: no voltage drop occurs in the bulk of the IL, which was verified by chronoamperometry, potential sweep measurements, and *BE* measurements at different XPS probing positions and with electrodes of different size (Figure S2–S12). Furthermore, no shifts or broadening of the F 1s XPS peak, caused by an ohmic potential drop, were found. Therefore, Δ*ϕ*_anodic_ can be directly converted to Δ*ϕ*_cathodic_ and vice versa because the applied voltage is identical to the sum of the two.

When the WE was grounded instead of the CE, the F 1s peak shifts as the amount of the potential screening at the WE, not at the CE; see Figure 1c (bottom). Therefore, Δ*BE* indicates Δ*ϕ*_anodic_ for a positive applied voltage and Δ*ϕ*_cathodic_ for a negative applied voltage.

Figure 1d shows Δ*BE* vs. the applied voltage with counter (black) and working (red) Pt electrodes grounded. For identical potential screening at both electrodes, defined as “ideal case”, the voltage drop at each electrode/IL interface would be half of the applied voltage and the F 1s peak would shift by half of the applied voltage. This ideal shift is plotted as dashed straight lines with ±0.5 eV/V slopes. From −3 to +3 V, Δ*BE* shows considerable deviations from the ideal line towards lower *BE*s, which indicates that for the IL/Pt interface Δ*ϕ*_anodic_ is larger than Δ*ϕ*_cathodic_. We attribute this asymmetric potential screening to the asymmetric structure/interaction of the EDL at the anode and cathode.

To further elucidate this behavior, we studied the interfaces of imidazolium‐based ILs and [C_4_C_1_Pyrr][Tf_2_N] with Pt and Au electrodes (see Table S1 in the SI). The corresponding Δ*ϕ*_cathodic_ are plotted in Figure 2a and 2b, respectively, as derived from the *BE* of the F 1s peaks; only for [C_8_C_1_Im]Cl, the N 1s peak was used. All measurements were performed within the electrochemical windows of the ILs; see chronoamperometry and potential sweep measurements in Figure S5–S12.

**Figure 2:**
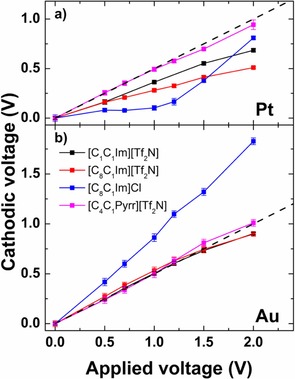
Cathodic voltage of various ILs vs. applied voltage. The ideal lines for equal potential drops at the anode and cathode interfaces are indicated as dashed straight lines (+0.5 V/V). a) Pt/Pt electrodes. b) Au/Au electrodes.

For Pt (Figure 2a), the Δ*ϕ*_cathodic_ values of all imidazolium‐based ILs are found below the ideal line, and thus are smaller than the Δ*ϕ*_anodic_ values. We propose that the strong bonding interaction between the π orbitals of imidazolium and Pt leads to stronger attraction of the cation to the cathode than of the anion to the anode. Therefore, the required potential for attracting cations to the cathode is smaller. For [C_4_C_1_Pyrr][Tf_2_N], the Δ*ϕ*_cathodic_ values nearly coincide with the ideal line, and thus are similar to Δ*ϕ*_anodic_, which is in line with the absence of specific π orbital interactions of the cation with the Pt electrodes.

For Au (Figure 2b), the Δ*ϕ*_cathodic_ values of the imidazolium‐based ILs with the large [Tf_2_N]^−^ anions and of [C_4_C_1_Pyrr][Tf_2_N] fall on the ideal line, which indicates similar potential screening on cathode and anode. This contrasts the situation with Pt and is attributed to the much weaker adsorption of aromatic molecules on Au than on Pt;^22^ thus, the asymmetric adsorption behavior of cation and anion likely does not occur on Au. A weak interaction of a similar IL, that is, [C_2_C_1_Im][Tf_2_N] with Au(111) has indeed been observed by AFM.^23^

In the absence of strong chemical interactions, the EDL capacitance is mainly influenced by the size of the counterion: in the Helmholtz model, the EDL capacitance is treated as a macroscopic capacitance, which is inversely proportional to the distance between two charged plates. For ILs, the distance between the charged plates can be assumed as the size of the counterions.11b For [C_1_C_1_Im][Tf_2_N], [C_8_C_1_Im][Tf_2_N], and $\left[C_{4}C_{1}\mathrm{Pyrr}\right]\left[{\mathrm{Tf}}_{2}N\right]$ , the size of [Tf_2_N]^−^ is similar to that of the cation. Thus, the EDL capacitance at anode and cathode might be similar for Au electrodes. The only exception from the generally observed behavior in Figure 2b is [C_8_C_1_Im]Cl, for which Δ*ϕ*_cathodic_ is much larger than Δ*ϕ*_anodic_, which is attributed to the much smaller size of Cl^−^ as compared to [Tf_2_N]^−^.

We derive Δ*ϕ* values by measuring changes in the IL *BE* positions with respect to the value at zero applied voltage. To study the effect of different ILs and electrodes on our reference point, we measured the F 1s XPS peak position at 0 V using both Pt and Au electrodes as well as different ILs. Notably, in all cases we found the same binding energy of 688.79 (±0.08) eV, which indicates very similar charging and potential screening at zero applied voltage (see Table S2 in the SI). Therefore, the observed large asymmetric potential screening effects described above are not due to the reference point employed.

Despite a number of experimental and modeling studies of multilayered structures at the IL/solid interface, the thickness of the EDL at the IL/electrode interface is still debated.11b Recent SFG^24^ and NMR^10^ results suggest that the counterions are localized in the first monolayer in contact with the charged surface. Within the IL electrochemical window, we found that the magnitude of potential screening strongly depends on the electrode material and the nature of the IL counterion. This implies that the observed potential screening is mainly governed by a single monolayer of counterions close to the electrode and not by counterions in multilayers.

For voltages around ±2.5 V and above, the deviation of Δ*BE* from the ideal line behavior starts to decrease for all studied ILs (Figure 2 and S5–S12) indicating equivalent potential screening on anode and cathode at high voltages. One possible explanation is that at around ±2.5 V, the amount of counterions in the EDL starts to exceed the coverage of one monolayer and thus potential screening at higher voltage is achieved by the counterions in multilayers. Likely, these further layers are not strongly influenced by the specific IL/electrode interaction, and thus the potential screening on anode and cathode becomes similar. A simple estimation based on the plate capacitor model (see the SI) shows that at 3 V the number of excess ions on the electrode roughly corresponds to 2 ML of IL, which supports our argument about the multilayer contribution at higher voltages.

While experimental studies of the potential screening under equilibrium conditions are scarce, asymmetric potential screening for the IL/electrode interfaces has been reported by molecular dynamics simulations.11d, 11e For example, the simulation for [C_2_C_1_Im][SCN] on a graphene electrode shows a smaller cathodic voltage, as is observed here.11c

In conclusion, we introduced a new approach to investigate the potential screening at various IL/electrode interfaces using *BE* shifts in in situ XPS measurements. We studied a variety of different ILs using a two‐electrode electrochemical cell with either identical Pt or Au electrodes. In the case of imidazolium‐based ILs and Pt electrodes, the potential screening at the anode is larger than at the cathode, which is attributed to strong interactions via specific adsorption of the imidazolium cations at Pt. In contrast, for Au electrodes in the absence of specific adsorption, no significant asymmetry is observed. Furthermore, the potential screening is affected by the thickness of the EDL, as is deduced from pronounced differences for small and large anions. Our results imply that at voltages up to ∼2 V the potential screening in ILs mainly occurs by counterions in the monolayer range on the electrode; only at higher voltages multilayers contribute. We are convinced that our approach provides a new route towards studying chemical interactions between ILs and electrodes by directly comparing experimental and simulation results. Our findings offer an excellent starting point for studying various IL/metal interfaces under ultraclean conditions.

## Experimental Section

The electrochemical cell developed by our group consists of a molybdenum sample holder and two metal wires as working and counter electrodes, which are connected to the potentiostat (Keithley 2450) through the contact pins of the head of the manipulator. Polytetrafluoroethene spacers are used to avoid the contact between sample holder and electrodes (see Figure S1). The Pt and Au wires (MaTeck), with diameters of 0.3 and 0.25 mm, have a purity grade of 99.99 % and 99.995 %, respectively, and were flame‐annealed prior to mounting. The [C_1_C_1_Im][Tf_2_N], [C8C1Im][Tf2N] , and [C_8_C_1_Im]Cl were synthesized under ultrapure conditions according to previous publications;20a, 20b [C_4_C_1_Pyrr][Tf_2_N] was purchased from Iolitech with a purity >99 %. The purities of ILs were carefully checked by XPS. For each combination of IL and metal, the electrode wires were carefully immersed into the IL in such way that their contact areas with the IL were identical (±5 %) to avoid geometry effects on the potential screening (see also discussion in the SI, Figure S4).

XPS was carried out using a monochromated Al K_α_ source and a hemispherical ARGUS analyzer. High‐resolution scans were recorded with a pass energy of 35 eV and a dwell time of 0.5 s with an overall energy resolution of 0.4 eV. Potential sweep measurements and chronoamperometry were performed using the Keithley 2450 source meter. The potential sweep measurement scan rate was 50 mV/s.

## Conflict of interest

The authors declare no conflict of interest.

## Supporting information

As a service to our authors and readers, this journal provides supporting information supplied by the authors. Such materials are peer reviewed and may be re‐organized for online delivery, but are not copy‐edited or typeset. Technical support issues arising from supporting information (other than missing files) should be addressed to the authors.

Supplementary
