# Supplementary material for: Potential Screening at Electrode/Ionic Liquid Interfaces from In Situ X‐ray Photoelectron Spectroscopy
Source: ChemistryOpen. 2019 Sep 19;8(12):1365–8. doi: 10.1002/open.201900211 (PMC6892450; doi:10.1002/open.201900211)
Supplement: Supplementary file 1 — Supplementary [file OPEN-8-1365-s001.pdf]

ChemistryOPEN Open Access

Supporting Information

© Copyright Wiley-VCH Verlag GmbH & Co. KGaA, 69451 Weinheim, 2019

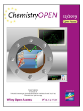

Potential Screening at Electrode/Ionic Liquid Interfaces from In Situ X-ray Photoelectron Spectroscopy

Francesco Greco+, Sunghwan Shin+,* Federico J. Williams, Bettina S. J. Heller, Florian Maier, and Hans-Peter Steinrück© 2019 The Authors. Published by Wiley-VCH Verlag GmbH & Co. KGaA. This is an open access article under the terms of the Creative Commons Attribution License, which permits use, distribution and reproduction in any medium, provided the original work is properly cited.

Supporting Information

Potential Screening at Electrode/Ionic Liquid Interfaces from In Situ X-ray Photoelectron Spectroscopy

Relation between the potential screening and the binding energy

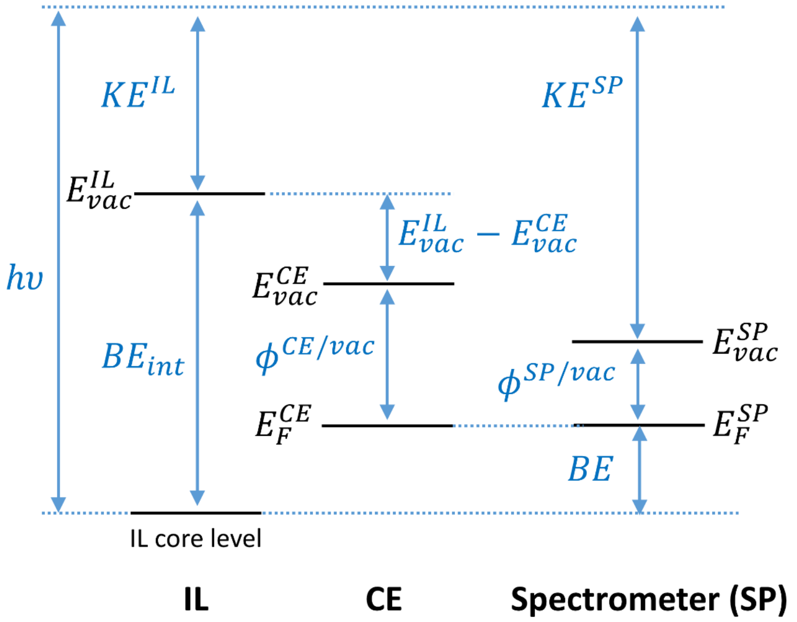

The external voltages were applied to the ionic liquids (ILs) through two identical (same materials and contact area) counter and working electrodes (CE and WE), of which one was connected to the ground potential together with the spectrometer of the X-ray photoelectron spectroscopy (XPS). In the case of the CE grounded, the intrinsic binding energy (BE_{int}) of the IL can be defined as

$$BE_{int} = hv - KE^{SP} - \phi^{SP/vac} + (E_{vac}^{IL} - E_{vac}^{CE}) + \phi^{CE/vac}$$

where hv is the photon energy, KE^{SP} is the kinetic energy of electrons at the spectrometer, $\phi^{SP/vac}$ is the work function of the spectrometer, $\phi^{CE/vac}$ is the work function of the CE and $E_{vac}^{IL} - E_{vac}^{CE}$ is the vacuum level difference between the IL and the CE. Note that, due to the difficulty to define the Fermi level of ILs, here we defined the BE_{int} of the IL as the energy difference between the core orbital level of atoms in the IL and the vacuum level of the IL like the convention of gas XPS.

Meanwhile, in this experiment, the binding energy (or measured binding energy, BE) was defined and reported based on the convention of a metallic sample measured by XPS as

$$BE = hv - KE^{SP} - \phi^{SP/vac}$$

and the BE is related to BE_{int} as

$$BE = BE_{int} - (E_{vac}^{IL} - E_{vac}^{CE}) - \phi^{CE/vac}$$

Note that BE_{int}, E_{vac}^{CE}, and $\phi^{CE/vac}$ are not affected by the applied potential, thus the BE is shifted as the amount of ΔE_{vac}^{IL}.

In the case of the CE grounded, the potential of the IL is shifted as the amount of the potential screening of CE ($e\varphi^{CE/IL}$) and the vacuum level difference between the IL and CE, $E_{vac}^{IL} - E_{vac}^{CE}$, is changed as the amount of this screening. This relation can be verified by the following cycle:

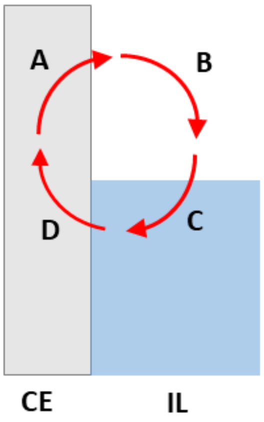

(A) an electron is brought from the Fermi level of the electrode to its vacuum level; this costs the work function $\phi^{CE/vac}$; (B) the electron is taken from the vacuum level of CE to that of the IL; this costs the difference in vacuum levels, $E_{vac}^{IL} - E_{vac}^{CE}$; (C) the electron is brought from the vacuum level of the IL to inside the IL, this costs the electrolyte surface potential (or the electric potential difference between the vacuum and the IL) $e\varphi^{vac/IL}$; (D) the electron is brought across the double layer to the metal Fermi level, this costs the electric potential difference between the IL and CE with the negative chemical potential of the electron in the metal ($e\varphi^{IL/CE} + \mu^{CE}$).

From this cycle, we can derive

$$\phi^{CE/vac} + (E_{vac}^{IL} - E_{vac}^{CE}) + e\varphi^{vac/IL} + e\varphi^{IL/CE} + \mu^{CE} = 0$$

Since $\phi^{CE/vac}$, $e\varphi^{vac/IL}$, and μ^{CE} are not depending on the applied potential, this implies that changes in the vacuum level difference between the electrode and the IL are equal to changes in the electrical double layer (EDL) potential:

$$\Delta(E_{vac}^{IL} - E_{vac}^{CE}) = e\Delta\varphi^{CE/IL}$$

Therefore, the shift of BE of the IL is proportional to the difference of the potential screening at the CE/IL interface.

$$\Delta BE = e\Delta\varphi^{CE/IL}$$

No ohmic drop in bulk IL

When the external potential (U) was applied between CE and WE electrodes, the electric potential inside the IL is expressed as,

$$U = (\mu^{WE} - \mu^{CE})/e + \left(\varphi^{WE/IL} + \varphi^{IL/CE}\right) + \Gamma$$

where U is the applied potential, μ^x is the chemical potential of the electron in a metal x, $\varphi^{x/IL}$ is the surface potential difference between the electrode x and the IL, and Γ is the ohmic drop in the bulk IL. When the potential was applied between two identical electrodes within the electrochemical window of the IL, the applied potential is simply equal to the sum of the potential screenings at both electrode interfaces.

$$U = \varphi^{CE/IL} + \varphi^{IL/WE}$$

In this study, when −2 to +2 V were applied, negligible ohmic drops were measured by chronoamperometry measurements (Figure S2) and XPS measurements performed on different probing positions (Figure S3).

A simple estimation of the density of excess charges at the IL/electrode interface

The amount of excess counterions at the IL/electrode interface can be estimated roughly based on a parallel plate capacitor model:

$$\rho = \frac{n}{A} = \frac{Q}{A * e} = \frac{C * V}{A * e} = \frac{\varepsilon * \varepsilon_0 * A}{d} * \frac{V}{A * e} = \frac{V * \varepsilon * \varepsilon_0}{e * d}$$

where ρ is the number density of excess charges (cm^{-2}), V is the potential screening on the electrode, e is the charge of an electron, d is the distance between two charged plates, ε is the dielectric constant of the IL at the IL/electrode interface, and ε_0 is the vacuum permittivity.

Here we assume the distance between plates as the size of ions (0.5 nm) and the dielectric constant of the IL as 5. According to this simple estimation, 5.5×10^{13} cm^{-2} excess counterions are needed to screen 1 V on the electrode and this coverage is 0.6 monolayer of IL. The surface density of IL on the electrode was estimated as 8.6×10^{13} ion pairs cm^{-2} from the 2D crystalline structure of [C_8C_1Im][Tf_2N] on Au(111). (B. Uhl et al. *Phys. Chem. Chem. Phys.*, **2015**, *17*, 23816) Thus the amount of excess charges can exceed the coverage of a monolayer at a higher

voltage than ±3 V.

Table S1. Cations and anions of ILs investigated in this study.

Abbreviation	Structure	Name
$[C_1C_1Im]^+$	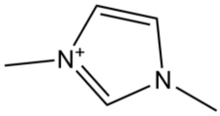	1,3-dimethylimidazolium
$[C_8C_1Im]^+$	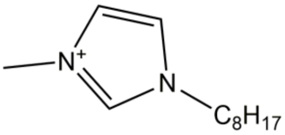	1-methyl-3-octylimidazolium
$[C_4C_1Pyrr]^+$	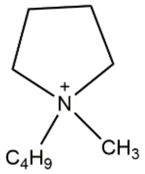	1-butyl-1-methylpyrrolidinium
$[Tf_2N]^-$	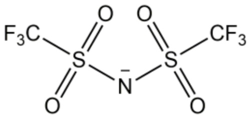	bis[(trifluoromethyl)sulfonyl]imide
Cl^-		chloride

Table S2. F 1s (or N 1s) peak position of various ILs at 0 V applied.

IL	BE with Pt electrodes (eV)	BE with Au electrodes (eV)
$[C_1C_1Im][Tf_2N]$	688.76	688.77
$[C_8C_1Im][Tf_2N]$	688.81	688.66
$[C_8C_1Im]Cl$	402.12	401.83
$[C_4C_1Pyrr][Tf_2N]$	688.91	688.83

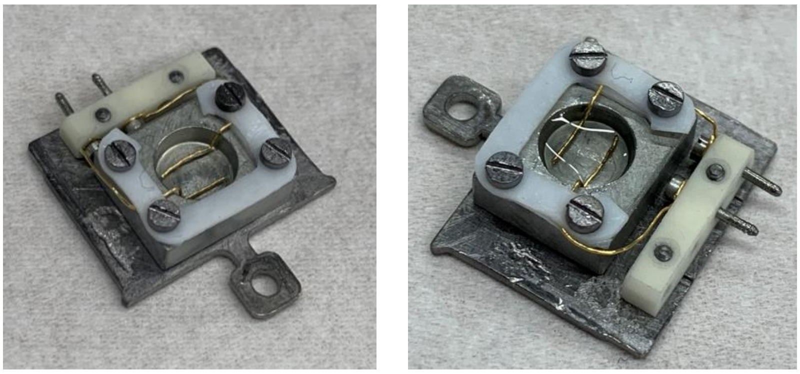

Figure S1. Electrochemical cell used for the potential screening measurements before filling IL (left) and after filling IL (right). The cell consists of: a molybdenum reservoir; metallic wire electrodes connected to molybdenum pins screwed in a ceramic; PTFE spacers in order to avoid contact between the electrodes and the reservoir.

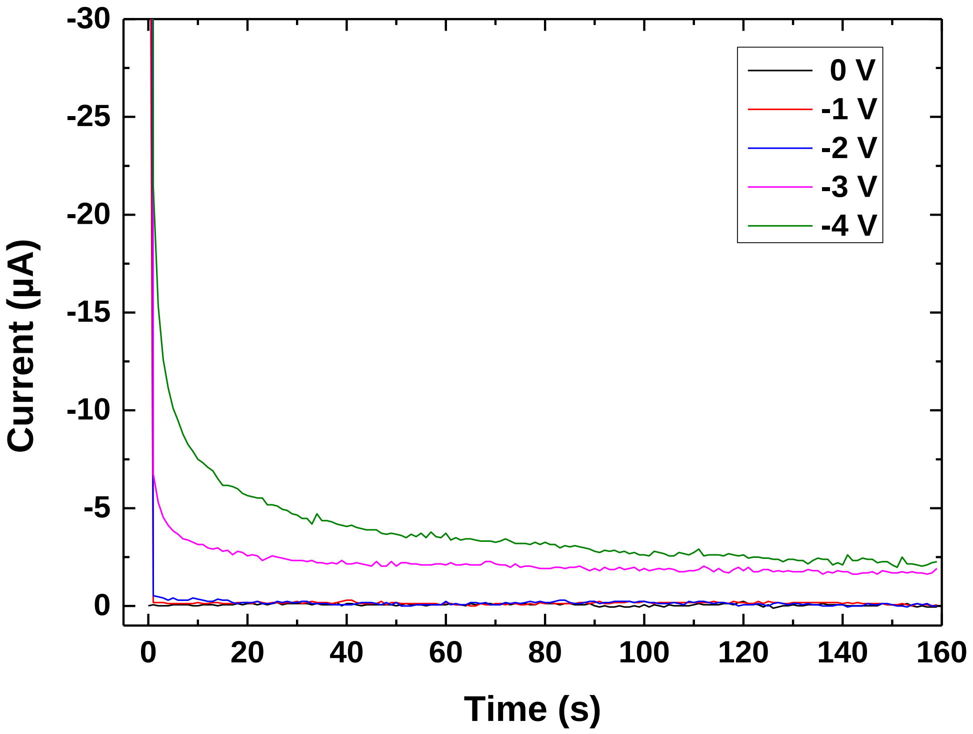

Figure S2. Current between Pt electrodes in [C_8C_1Im][Tf_2N] with a constant applied potential. In the cases of 0, −1, and −2 V, the charging current for an EDL formation decreased rapidly within 1 s. XP spectra were measured for 150 s approximately after the charging process and a residual current during the XPS measurement was less than 0.1 μA. This small residual current implies that an ohmic drop between the electrodes was negligible.

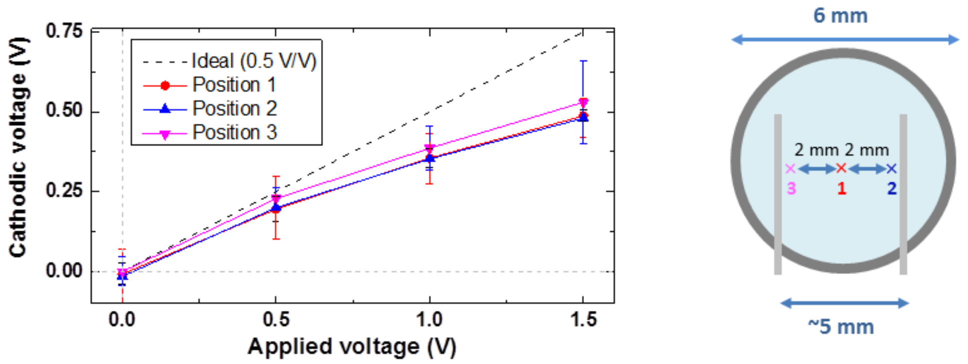

Figure S3. (Left) Cathodic voltage of [C_1C_1Im][Tf_2N] at different probing positions between Pt electrodes. (Right) Schematic sketch of XPS probing positions. In case of an ohmic drop between the electrodes, the potential of the IL should change depending on the position, with the XPS peak shifting accordingly. Our data show that the XP peaks did not change depending on the probing positions, which implies that no ohmic drop exists between the electrodes.

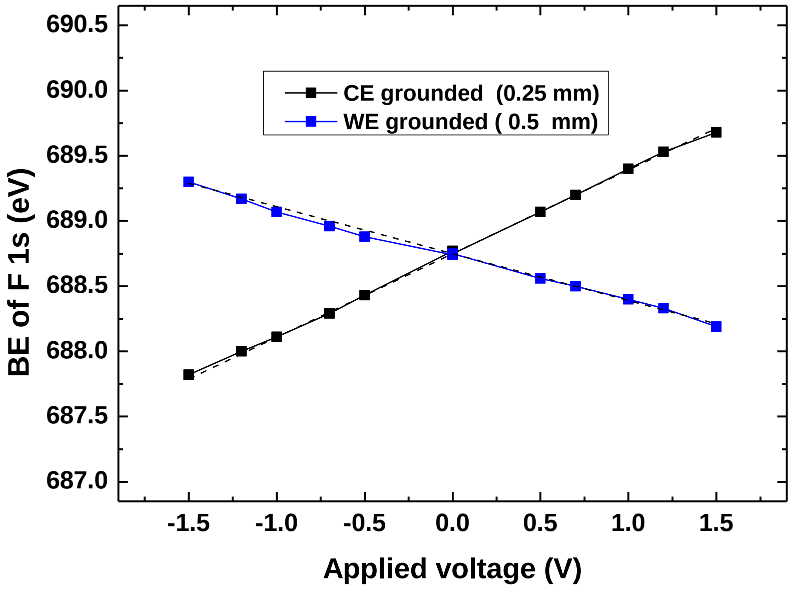

Figure S4. The binding energy of [C_1C_1Im][Tf_2N] with different size Au electrodes. CE: 0.25 mm diameter, WE: 0.5 mm diameter. The linear fittings are indicated as dashed lines with a 0.64 eV/V slope for CE grounded and a −0.36 eV/V for WE grounded. According to a parallel plate capacitor model, the capacitance of the EDL is proportional to the surface area of the electrode and the potential screening is inversely proportional to the surface area of the electrodes. The expected slopes from this model are 0.67 eV/V and −0.33 eV/V, respectively. Our results coincide with the predicted values within less than 10% error.

<u>**BE shifts, residual current and potential sweep of various ILs**</u>

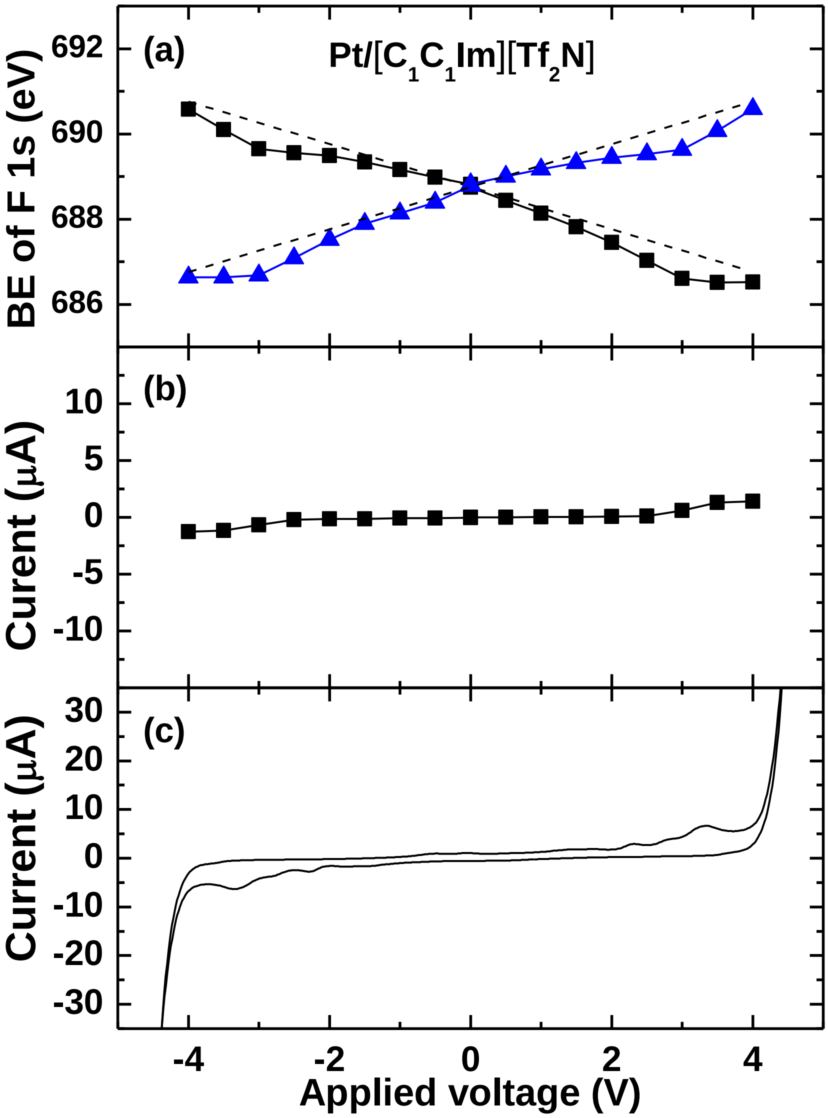

Figure S5. (a) The binding energy shift of F 1s in $[C_1C_1Im][Tf_2N]$ XP spectra according to an applied external potential in the cases of a Pt WE-grounded (d=0.3 mm) (black square) and a Pt CE-grounded (d=0.3 mm) (blue triangle) setups. The ideal lines are indicated as dashed lines. (b) Residual current at a constant applied potential during the XPS measurement. (c) Potential sweep measurement. The ramping rate is 50 mV/s.

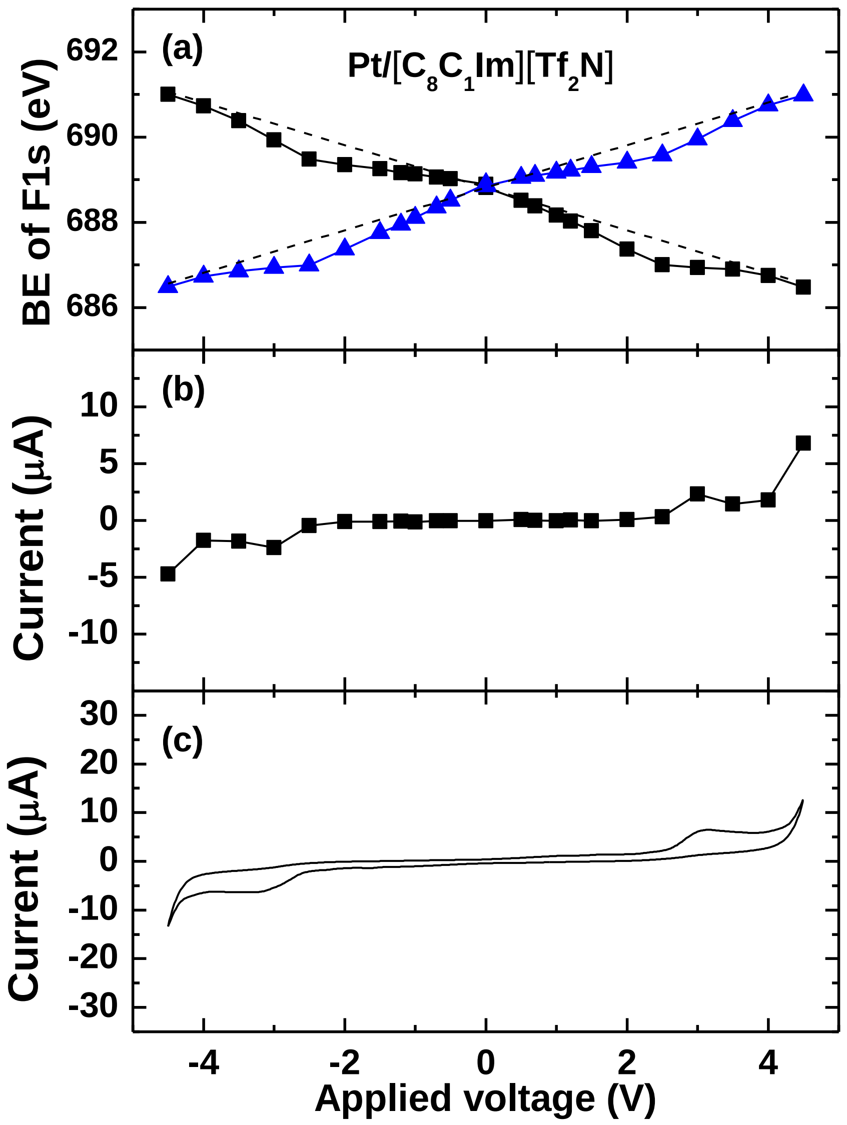

Figure S6. (a) The binding energy shift of F 1s in $[C_8C_1Im][Tf_2N]$ XP spectra according to an applied external potential in the cases of a Pt WE-grounded (d=0.3 mm) (black square) and a Pt CE-grounded (d=0.3 mm) (blue triangle) setups. The ideal lines are indicated as dashed lines. (b) Residual current at a constant applied potential during the XPS measurement. (c) Potential sweep measurement. The ramping rate is 50 mV/s.

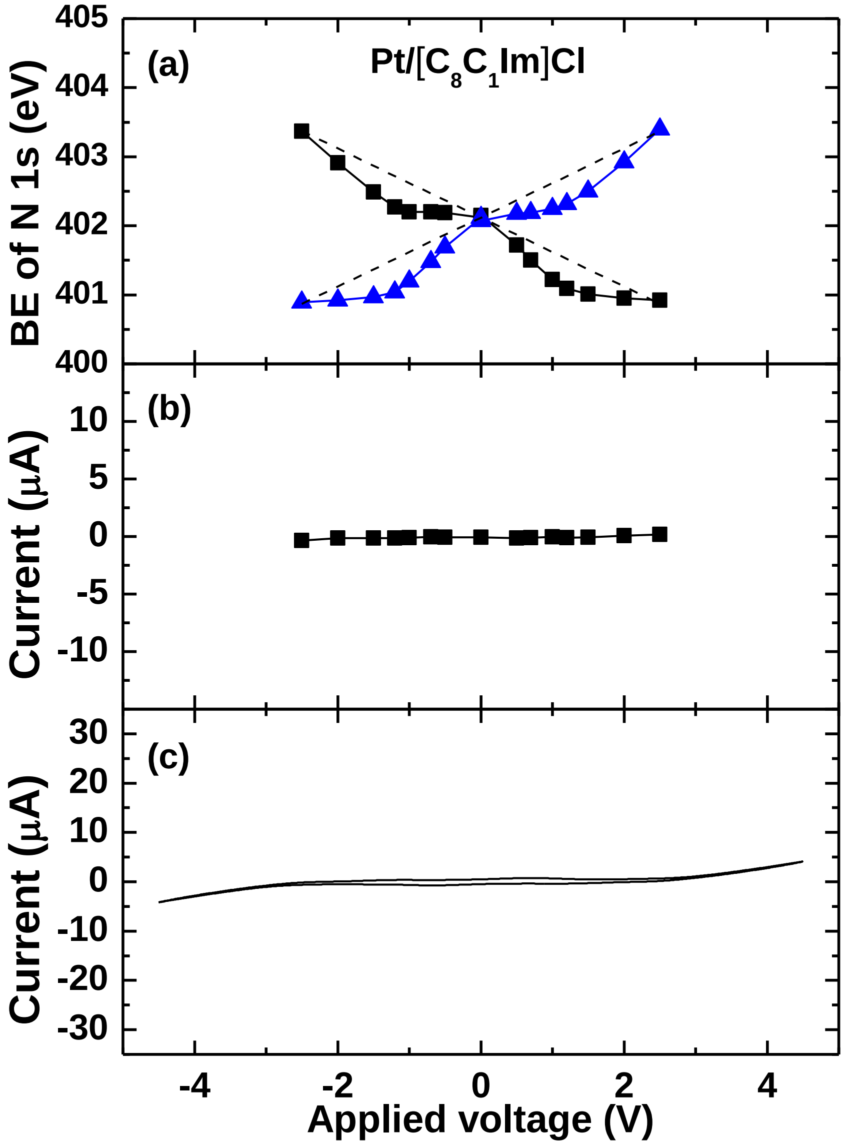

Figure S7. (a) The binding energy shift of N 1s in $[C_8C_1Im]Cl$ XP spectra according to an applied external potential in the cases of a Pt WE-grounded (d=0.3 mm) (black square) and a Pt CE-grounded (d=0.3 mm) (blue triangle) setups. The ideal lines are indicated as dashed lines. (b) Residual current at a constant applied potential during the XPS measurement. (c) Potential sweep measurement. The ramping rate is 50 mV/s.

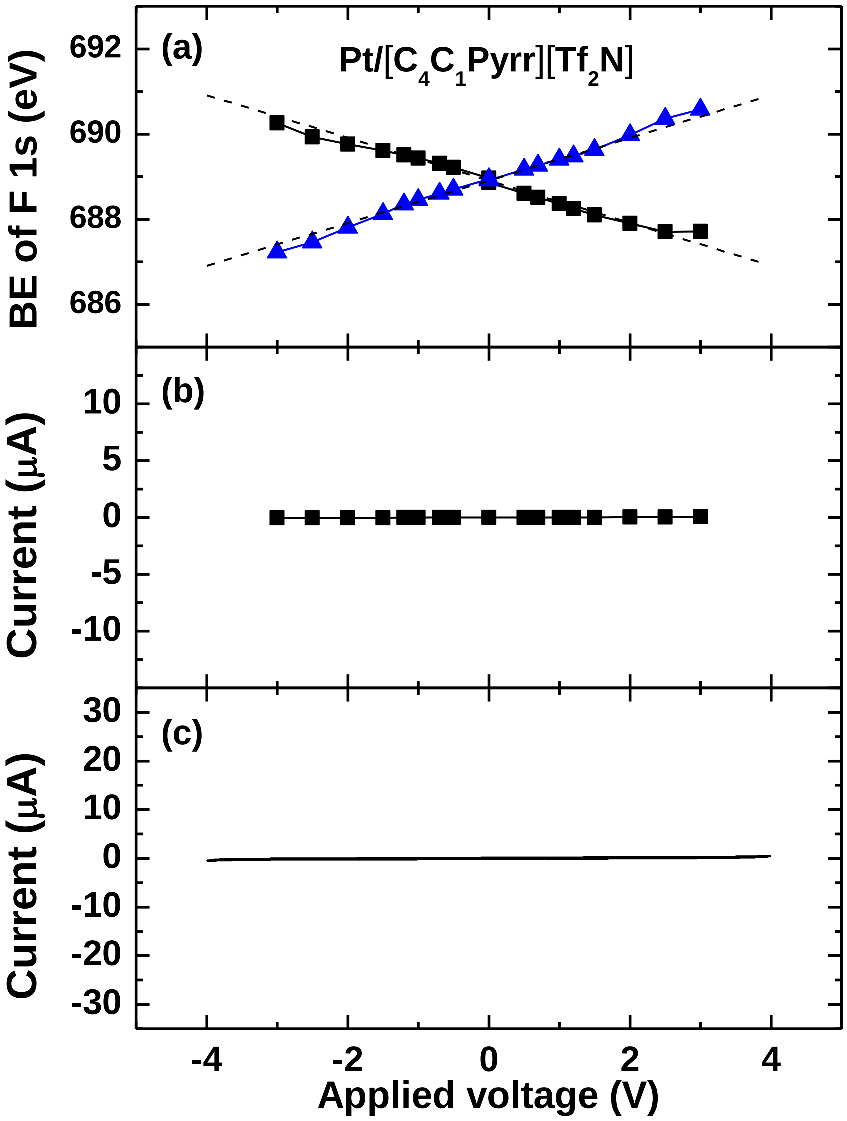

Figure S8. (a) The binding energy shift of F 1s in [C_4C_1Pyrr][Tf_2N] XP spectra according to an applied external potential in the cases of a Pt WE-grounded (d=0.3 mm) (black square) and a Pt CE-grounded (d=0.3 mm) (blue triangle) setups. The ideal lines are indicated as dashed lines. (b) Residual current at a constant applied potential during the XPS measurement. (c) Potential sweep measurement. The ramping rate is 50 mV/s.

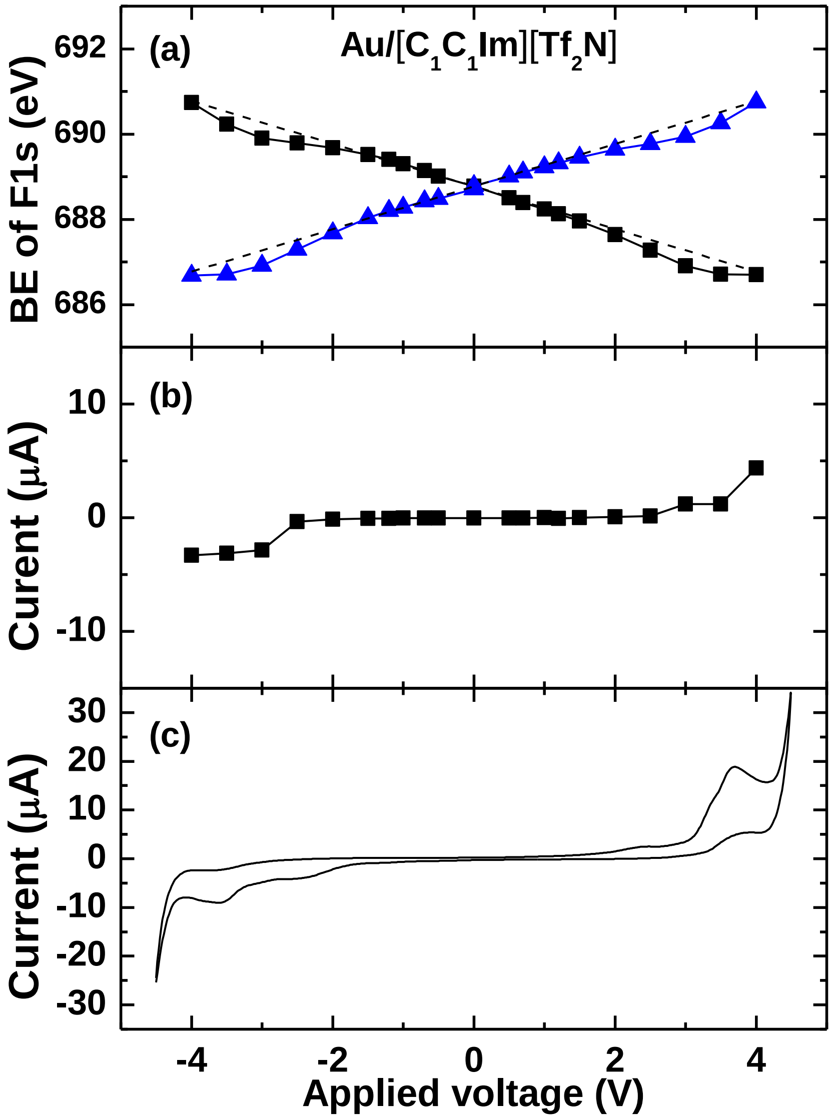

Figure S9. (a) The binding energy shift of F 1s in $[C_1C_1Im][Tf_2N]$ XP spectra according to an applied external potential in the cases of an Au WE-grounded (d=0.25 mm) (black square) and an Au CE-grounded (d=0.25 mm) (blue triangle) setups. The ideal lines are indicated as dashed lines. (b) Residual current at a constant applied potential during the XPS measurement. (c) Potential sweep measurement. The ramping rate is 50 mV/s.

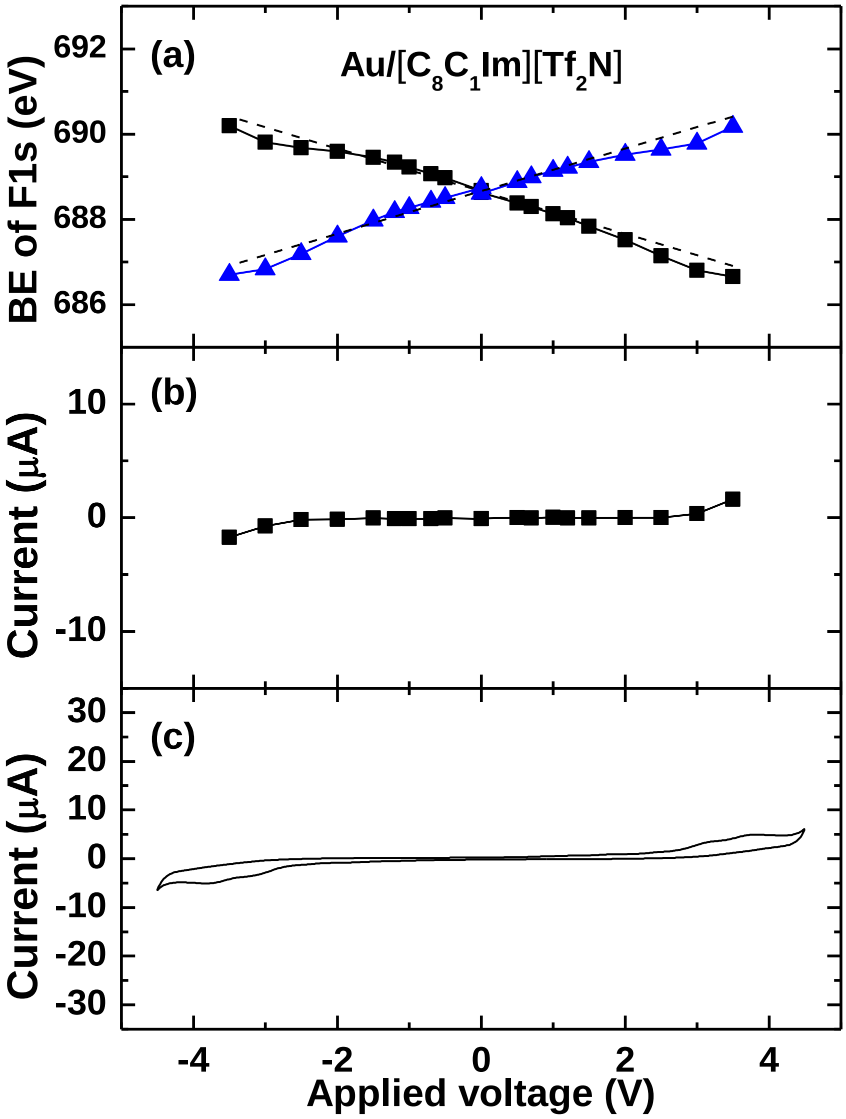

Figure S10. (a) The binding energy shift of F 1s in $[C_8C_1Im][Tf_2N]$ XP spectra according to an applied external potential in the cases of an Au WE-grounded (d=0.25 mm) (black square) and an Au CE-grounded (d=0.25 mm) (blue triangle) setups. The ideal lines are indicated as dashed lines. (b) Residual current at a constant applied potential during the XPS measurement. (c) Potential sweep measurement. The ramping rate is 50 mV/s.

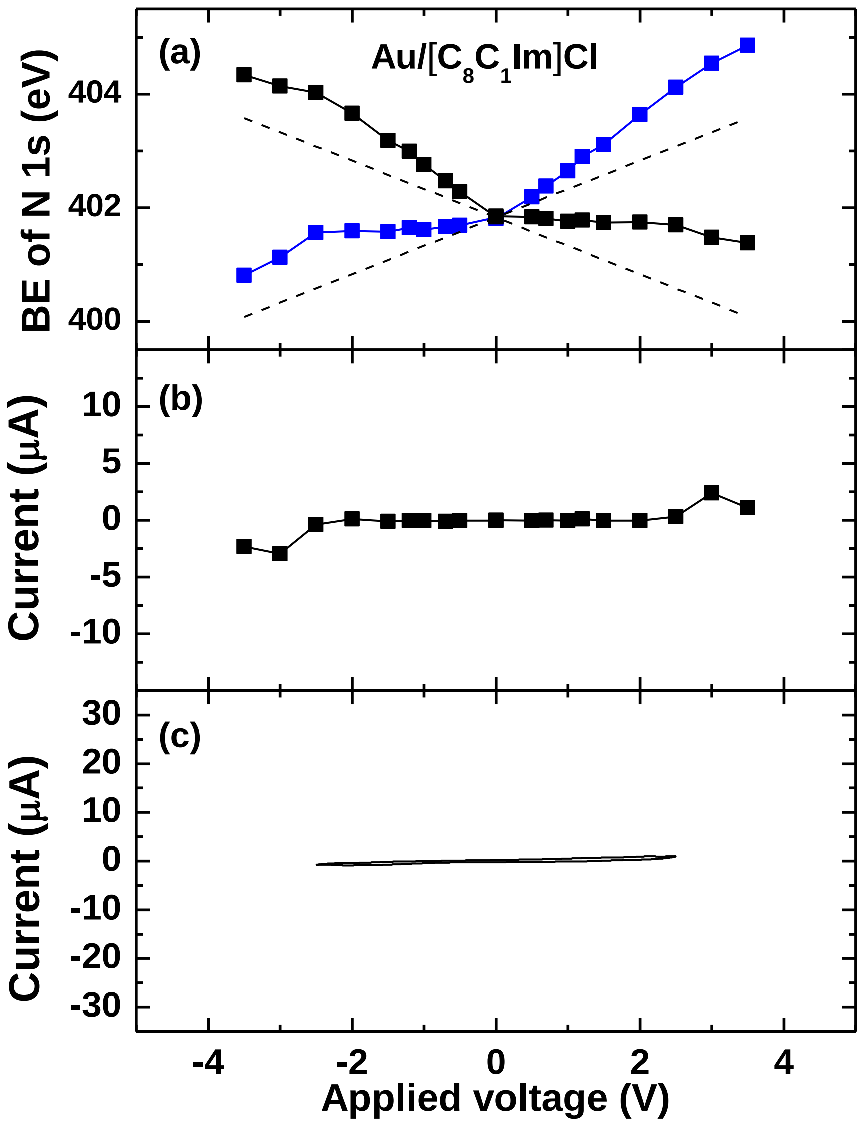

Figure S11. (a) The binding energy shift of N 1s in $[C_8C_1Im]Cl$ XP spectra according to an applied external potential in the cases of an Au WE-grounded (d=0.25 mm) (black square) and an Au CE-grounded (d=0.25 mm) (blue triangle) setups. The ideal lines are indicated as dashed lines. (b) Residual current at a constant applied potential during the XPS measurement. (c) Potential sweep measurement. The ramping rate is 50 mV/s.

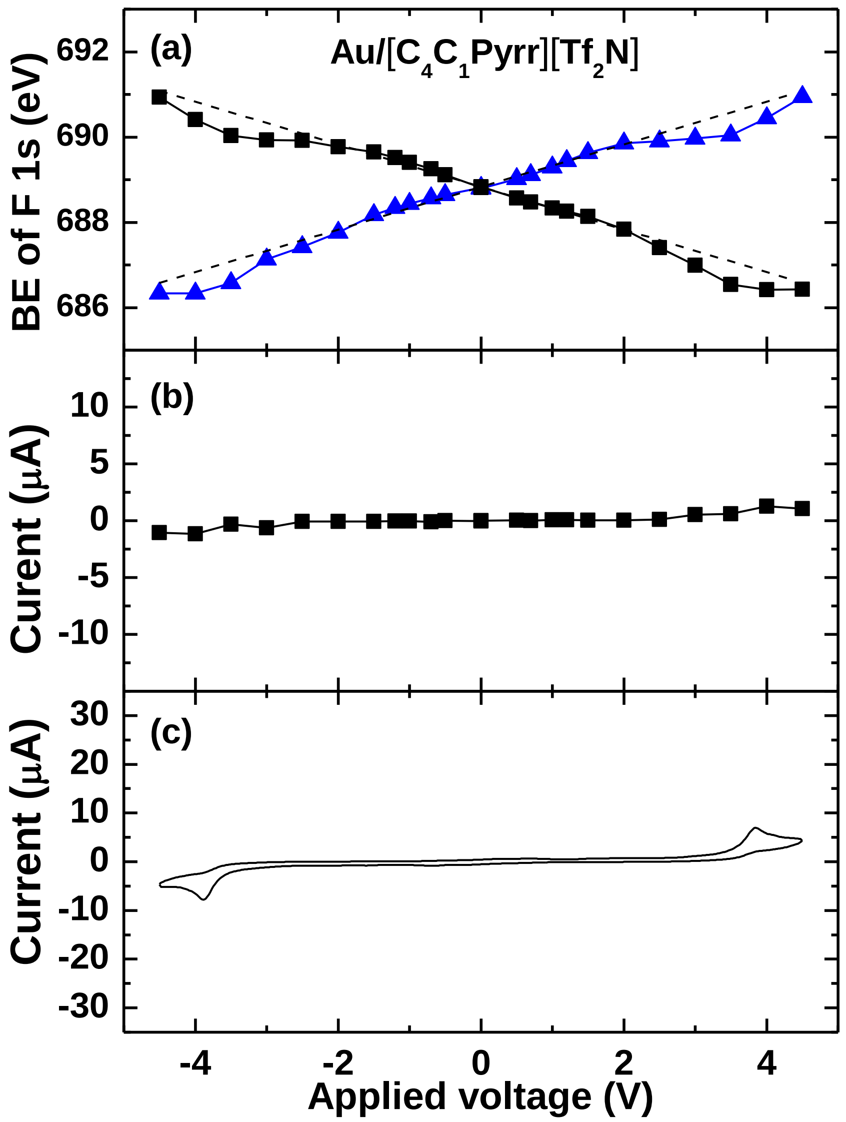

Figure S12. (a) The binding energy shift of F 1s in $[C_4C_1Pyrr][Tf_2N]$ XP spectra according to an applied external potential in the cases of an Au WE-grounded (d=0.25 mm) (black square) and an Au CE-grounded (d=0.25 mm) (blue triangle) setups. The ideal lines are indicated as dashed lines. (b) Residual current at a constant applied potential during the XPS measurement. (c) Potential sweep measurement. The ramping rate is 50 mV/s.
